# Process Evaluation of Teaching Critical Thinking About Health Using the Informed Health Choices Intervention in Kenya: A Mixed Methods Study

**DOI:** 10.9745/GHSP-D-23-00485

**Published:** 2024-12-20

**Authors:** Faith Chesire, Andrew D. Oxman, Margaret Kaseje, Violet Gisore, Michael Mugisha, Ronald Ssenyonga, Matt Oxman, Allen Nsangi, Daniel Semakula, Laetitia Nyirazinyoye, Nelson K. Sewankambo, Heather Munthe-Kaas, Christine Holst, Sarah Rosenbaum, Simon Lewin

**Affiliations:** a Tropical Institute of Community Health and Development, Kisumu, Kenya.; b Institute of Health and Society, Faculty of Medicine, University of Oslo, Norway.; c Centre for Epidemic Interventions Research, Norwegian Institute of Public Health, Oslo, Norway.; d School of Public Health, College of Medicine and Health Sciences, University of Rwanda, Kigali, Rwanda.; e Makerere University, College of Health Sciences, Kampala, Uganda.; f Faculty of Health Sciences, Oslo Metropolitan University, Oslo, Norway.; g Department of Health Sciences Ålesund, Norwegian University of Science and Technology, Ålesund, Norway.; h Health Systems Research Unit, South African Medical Research Council, Cape Town, South Africa.

## Abstract

**Introduction:**

We evaluated the Informed Health Choices secondary school intervention to help students in Kenya think critically about health choices. We conducted this process evaluation to explore if the intervention was implemented as planned, identify factors that facilitated or hindered implementation, potential benefits of the intervention, and how to scale up the intervention beyond the trial.

**Methods:**

This was a mixed methods process evaluation nested in a cluster-randomized trial of the Informed Health Choices intervention. We analyzed quantitative data from teacher training evaluation forms completed by 39 teachers, 10 lesson evaluation forms completed by 40 teachers allocated to the intervention, and 72 structured classroom observation forms. We conducted a framework analysis of qualitative data from 14 group interviews (with 96 students, 23 teachers, and 18 parents) and 22 individual interviews (with 8 teachers, 5 school principals, 6 curriculum developers, and 3 policymakers). We assessed confidence in our findings from the qualitative analysis using a modified version of Confidence in the Evidence from Reviews of Qualitative Research.

**Results:**

Lesson objectives were achieved with minimal adaptations. Factors that might have facilitated the implementation of the intervention include teacher training; perceived value of the intervention by students, teachers, and policymakers; and support from school administration. Time constraints, teachers’ heavy workloads, and the lessons not being included in the curriculum or national examination are factors that might have impeded implementation. Both students and teachers demonstrated the ability to apply key concepts that were taught to health choices and other choices. However, they experienced difficulties with 2 of the lessons.

**Conclusion:**

Scale-up of this intervention in Kenyan schools is feasible but may depend on adjusting the time allocated to teaching the lessons, modifying the 2 lessons that teachers and students found difficult, and including the lesson objectives and assessment in the national curriculum.

## Abstract (precis)

Factors that facilitated the implementation of the Informed Health Choices intervention included the teacher’s training workshop, the perceived value of the intervention by multiple stakeholders, and support from education officials and school management.

## Teaser

**Key Findings:**

- The Informed Health Choices secondary school intervention was largely implemented as planned, but extended time for teaching and the use of Swahili were needed.
- Factors that facilitated the effective implementation included teacher training; perceived value of the intervention by students, teachers, and policymakers; and support from education officials, school managers, and administration. Time constraints, heavy workloads, and the lessons not being included in the curriculum are factors that may have adversely affected implementation of the intervention.
- Both teachers and students were able to recognize claims about the effects of health actions, assess them, and make informed choices about what to believe and do.

**Key Implication:**

- Scale-up of the intervention in Kenya is feasible but may depend on adjusting the time allocated to teaching the lessons, revision of the 2 lessons that teachers and students found difficult, teaching the difficult lessons toward the end of the secondary school education, and including lesson objectives in the national curriculum and teacher training.

See related articles by Mugisha et al. and Ssenyonga et al.

## BACKGROUND

Every day, we make many health-related decisions based on information from various sources, including social and mass media, which often present claims about what may improve or harm our health. Unfortunately, much of this information, especially in the media, is unreliable.^1^ Many adults and young people lack the skills to assess the health claims that they encounter.^2^^–^^5^ Acting on unreliable health claims or choosing not to act on reliable ones can result in unnecessary suffering or a waste of resources. This is especially a problem in low-income countries, where people have few resources. Developing critical thinking skills is essential for individuals to evaluate health information and make informed choices.^6^ Teaching these skills to young people can lay a foundation for future learning.

Globally, critical thinking is a key educational goal.^7^^,^^8^ In Kenya, for example, a competency-based curriculum has been adopted that includes critical thinking as a core competency. However, critical thinking about health is not explicitly included in the curriculum in Kenyan schools^9^ nor in other countries.^10^^–^^12^ Studies have shown that educational interventions aimed at improving people’s ability to assess health claims are effective, at least in the short term.^13^

To address this problem, the Informed Health Choices (IHC) Key Concepts framework identified 49 concepts that people should understand and apply when deciding whether to believe a claim about the effects of health actions (things that people do to care for their health or the health of others) and what decisions to make about their health.^14^^,^^15^ Between 2013 and 2017, we developed the IHC primary school intervention to teach primary school children,^16^ which was evaluated in a cluster-randomized trial in Uganda. The trial showed the intervention had a large positive effect on the ability of children to assess health claims and make informed health choices. A process evaluation alongside the trial found that the intervention was useful, but barriers to scaling up the intervention included a lack of time in the curriculum and cost of printing the resources.^18^

Building on the findings from the primary school trial and process evaluation in Uganda and context analyses conducted in Kenya, Rwanda, and Uganda,^9^^–^^11^^,^^17^^,^^18^ we developed the IHC secondary school intervention.^17^ The intervention included teacher training and digital resources for 10 lessons, focusing on 9 prioritized IHC key concepts (Box 1).^19^ Between 2020 and 2022, we worked closely with teachers, students, and curriculum developers, using a human-centered design approach to develop the intervention.^20^ Details of the IHC intervention are described elsewhere in the randomized cluster trial protocol, summarized in Box 2, with further information in the GREET checklist (Supplement 1).^21^

BOX 1 Key Concepts Included in the Informed Health Choices Secondary School Intervention

1. Treatments can cause harms as well as benefits.
2. Large, dramatic effects are rare.
3. Personal experiences or anecdotes alone are an unreliable basis for most claims.
4. Treatments that are new or technologically impressive may not be better than available alternatives.
5. Widely used treatments or those that have been used for decades are not necessarily beneficial or safe.
6. Identifying the effects of treatments depends on making comparisons.
7. Small studies may be misleading.
8. Comparison groups should be as similar as possible.
9. Weigh the benefits and savings against the harms and costs of acting or not.

BOX 2 Summary of the Informed Health Choices Secondary School Intervention

**Goal:** To give secondary school students a basic ability to think critically about health actions (things that people do to care for their health or the health of others); understand why thinking critically is important; to be able to recognize claims about the effects of health actions; and assess some of those claims.

**Underlying theory:** We used the Informed Health Choices (IHC) key concepts framework as a starting point for this educational intervention. These concepts or principles are intended to improve people’s ability to make better decisions on what to believe and do when faced with health claims and choices. The framework is based on evidence of the importance of the included concepts, logic, feedback, and other relevant framework.

**Content and planned delivery:** The intervention included:

- 2–3-day training workshop for secondary school teachers, facilitated by other teachers, to introduce them to the IHC learning resources and learning content.
- 10 lessons for students in a single school term, with each lesson taught in 40 minutes.
- Overview and background for each of the 10 lessons for teachers. The 10 lessons were delivered by the teachers during regular classroom time or, if necessary, outside of regular classroom time. They could use a computer, smartphone, or printouts to support delivery of the lessons.

**Delivery:** Depending on what equipment was available to the teachers, teachers delivered the lessons to students using a data projector and slide presentations that are included in the digital resources. In case of a power cut, teachers were provided with a blackboard-based lessons. The number of students in a class varied, and teachers used teaching strategies such as guided note- taking, small group discussion, use of response cards, homework, use of a standard lesson structure, setting objectives, and providing feedback.

We conducted a cluster-randomized trial to evaluate the effect of the intervention, enrolling 3,362 students across 80 secondary schools in Kenya.^21^ The trial compared students in schools implementing the intervention to those following the standard curriculum. The primary outcome was the proportion of students achieving a passing score on the Critical Thinking about Health Test, a test designed to measure their critical thinking skills in evaluating health-related choices. Results indicated a substantial effect of the intervention, with 62% of year 1 students in the intervention schools achieving a passing score on the Critical Thinking about Health Test, compared to 34% in the control schools.

We conducted this process evaluation alongside the trial to explore: (1) the extent to which the IHC secondary school intervention was implemented as planned, (2) factors that potentially facilitated or hindered effective implementation and might affect effective scale-up in Kenya, and (3) other potential benefits of the intervention, besides those assessed in the trial. In a separate study, we are exploring potential adverse effects.^14^

We conducted this process evaluation alongside the trial to explore the extent to which the IHC secondary school intervention was implemented as planned and factors that potentially facilitated or hindered effective implementation and might affect effective scale-up in Kenya.

## METHODS

**FIGURE 1:**
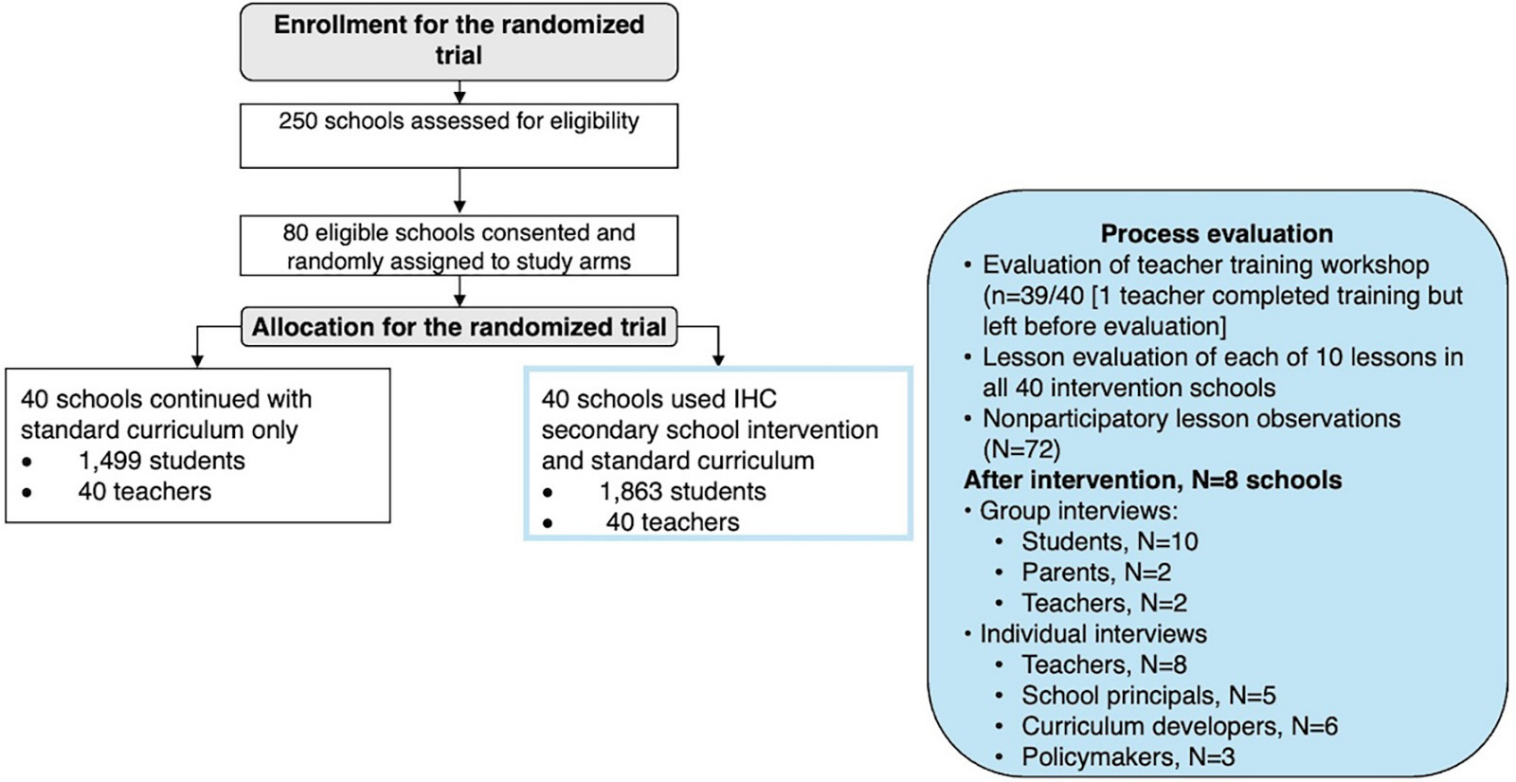
Process Evaluation Conducted as Part of the Intervention Arm of a Cluster-Randomized Trial of the IHC Secondary School Intervention, Kenya Abbreviation: IHC, Informed Health Choices.

**FIGURE 2:**
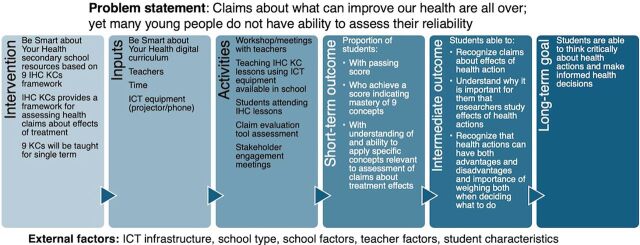
Logic Model of the IHC Secondary School Intervention, Kenya Abbreviations: ICT, information communication technology, IHC, Informed Health Choices, KC, key concept.

This was a mixed methods process evaluation nested in a cluster-randomized trial (Figure 1) using both qualitative and quantitative methods approach to data collection. We used a logic model to link the process evaluation findings to the trial findings (Figure 2).

### Study Setting and Location

This process evaluation was conducted in 40 secondary schools from 5 subcounties in Kisumu County that were assigned to the intervention arm of the trial. Secondary schools in Kenya follow the Kenya National Secondary School curriculum, which lasts for 4 years. An annual academic calendar is organized in 3 school terms, where a single term is 10–13 weeks. At the end of each term, students sit for an end-of-term examination to assess their understanding of topics taught in each subject during the period.

Teachers at this level are trained and accredited by a university or a teacher training institute. Nearly 95% of secondary school teachers hold a bachelor’s degree, and about 5% have a post-graduate qualification.^23^ Students enroll in lower secondary school when they are aged 13–14 years. Students in lower secondary schools are expected to choose 12 of 30 subjects. Currently, health and critical thinking are taught across subjects but not specifically critical thinking about health.^9^

### Sampling and Recruitment

We collected quantitative data from all 40 intervention schools. For the qualitative component of the evaluation, we purposively selected 8 schools based on geographical location (rural/urban) and ownership (private/public). We tried to ensure equal representation in the sample of schools according to the version (projector and blackboard) used to deliver the lessons.

In each of the 8 selected schools, we recruited the principal, the teacher who taught the IHC lessons, 12 students, and the parents of those students. Together with the teachers, we sampled the 12 students based on their performance at end-of-term examinations (high, moderate, and low) and sex. The participants are summarized in Table 1.

**TABLE 1.** Summary of Participants in Informed Health Choices Intervention Lesson Observations, Kenya

**Data Collection Method**	**Participant**	**No.**
Post-training evaluation	Teachers	39
Lesson evaluations	Teachers	40 for each of 10 lessons delivered
Nonparticipatory lesson observation	Teachers and students	All 10 lessons observed in all (n=40) schools at least once and more than 4 times in 8 selected schools
Focus group discussions	Students	10
	Teachers	2
	Parents	2
Individual interviews	School principals	5
	Policymakers in education officers	3
	Curriculum developers	6
	Teachers	8

We included all parents of the sampled students. Through the principals, we contacted those parents by phone and invited them to participate in interviews. In addition, we included officers from the county Ministry of Education, the Teachers’ Service Commission, and the Kenya Institute of Curriculum Development who were aware of the intervention. We contacted their offices by phone or letter, seeking permission to interview them.

### Data Collection

We collected qualitative data via nonparticipatory structured observations of lessons and interviews. We interviewed students, their parents, and policymakers individually. We interviewed some teachers individually and some in groups. Using a structured observation form, the principal investigator (FC) and trained research assistants observed lessons without participating in any classroom activities. FC led the interviews while a research assistant took notes. All the interviews and discussions were conducted in English except for some discussions with students and parents, which were done in Swahili or Luo. We took audio-recordings of the interviews and transcribed and translated the files verbatim. We de-identified all scripts before analysis.

For quantitative data, we used forms for evaluation of the teacher-training by teachers, evaluation of each lesson by teachers, and structured classroom observations. Both the teacher training evaluations and lesson evaluations were collected using electronic forms. We exported the data to Excel and generated descriptive analyses.

### Qualitative Data Analysis

We analyzed data for each objective. For qualitative data, we used a framework analysis approach. Two investigators (FC and VG) familiarized themselves with the data. They independently read and re-read the transcripts, identified relevant statements and codes guided by the frameworks (Supplement 2), and used inductive themes emerging from the data. They coded all transcripts and discussed and agreed on the final codes.

We then extracted coded statements from the scripts and charted the data by writing a summary of the findings for each framework factor. All the data was analyzed with the aid of N-vivo software. We compared coded data for each framework factor across all the data sources to obtain a deeper understanding of the meaning and implications of the results. Throughout the analysis, all findings were discussed within the research group (MK, ADO, SR, and SL) to improve the consistency and accuracy of the coding and the interpretation. The team discussed definitions of factors in the frameworks, resolved divergent views, and revised them in line with the codes and categories that emerged from the data.

### Quantitative Data Analysis

We used SPSS to perform descriptive analyses of the quantitative data from the training evaluation and lesson evaluation forms. The forms included 5-point Likert-scale items (1- “not confident,” 5- “very confident”) for teachers to rate the workshop and their level of confidence in teaching the lessons (from “very unprepared’’ to “very prepared’’).

#### Confidence in the Findings

We assessed confidence in the findings using a modified version of the Grading of Recommendations Assessment, Development, and Evaluation for Confidence in the Evidence from Reviews of Qualitative research (GRADE-CERQual) approach.^24^ GRADE-CERQual is a systematic and transparent method for assessing confidence in evidence from reviews of qualitative research based on 4 components: methodological limitations, data adequacy, coherence, and relevance.^25^ Although GRADE-CERQual has been designed for findings emerging from syntheses of qualitative evidence, the components of the approach are suitable for assessing findings from a single study with multiple sources of qualitative data.^24^ We rated confidence in each finding as being high, moderate, or low (Supplement 3). All findings started as high confidence and were downgraded if there were any important concerns for each of the 4 components.

### Reflexivity

As part of the research team, we engaged in a reflexivity process to critically reflect on our role and expectations. This began with individual written reflections, where each team member considered their expectations of the process evaluation findings and how their personal background and experience might influence their approach to the evaluations or their interpretation of the results. Following this, we held 2 team reflexivity discussions. These discussions highlighted key themes, including the intervention’s effects, project sustainability and scalability, the scope of the evaluation, the relationship between researchers and participants, and dynamics within the research team. These reflexive insights informed both our analysis and the writing of the process evaluation. A detailed description of the reflexivity process and the emerging themes can be found in Supplement 4.

### Ethical Approval

This study was approved and licensed in 2019 by the National Commission of Science and Technology under license number NACOSTI/P/19/1986. Further approvals were obtained from the Ministry of Education and Teachers Service Commission both at national and county levels. All participants older than age 18 years gave written consent to participate in the study. Each principal consented in loco parentis on behalf of their students (17 years and younger). In Kenya, school principals and school management boards have a guardianship role in schools and make decisions on behalf of their students. All students gave their assent to participate in interviews.

## FINDINGS

### Extent to Which the Intervention Was Delivered as Intended

#### Time Used to Prepare for the Lessons

The lesson evaluation data showed that teachers spent, on average, 15–30 minutes preparing for a lesson. As shown in Table 2, the shortest average preparation time for a lesson was 15.3 minutes, while the longest was 30.4 minutes. Most teachers cited competing priorities, heavy workloads, and prioritization of examinable subjects over nonexaminable subjects as factors that hindered preparation (high confidence).

**TABLE 2.** Time Used for Lesson Preparation and Teaching in Informed Health Choices Intervention Lesson Observations, Kenya

	**Mean Time to Prepare Lesson, Minutes**	**Mean Time to Complete the Lesson, Minutes**
1. Health actions	30.2	68.2
2. Health claims	30.4	50.4
3. Unreliable claims	15.3	62.7
4. Reliable claims	27.8	82.6
5. Using what we learned (1)	24.4	50.5
6. Randomly created groups	29.7	120.7
7. Large-enough groups	30.2	113.2
8. Personal choices	27.5	80.6
9. Community choices	21.3	50.6
10. Using what we learned (2)	24.4	60.9

#### Time Used to Teach the Lessons

According to the lesson evaluation data, it took an average of 74 minutes for delivery of each lesson compared to the suggested 40 minutes per lesson. Eight of 10 lessons took between 50.4 and 82.6 minutes, while it took on average 120.7 minutes for the lesson on “randomly created groups” and 113.2 minutes for the lesson on “large-enough groups” (Table 2). Based on teacher interviews, the extended lesson durations of more than the suggested 40 minutes were due to the embedded class activities (such as role play and group discussions) and the need for repeated clarifications sought by students on some concepts.

To accommodate this extra time, teachers sought permission from the school administration to reduce or forgo other school activities, such as physical education, games, and revision (study) time (high confidence).

*When it comes to time, there’s no single lesson I did [in] less than 1 hour. You know the issue is, when it comes to examples, the students will give the examples, you will discuss the examples, and it needs time. So, most of my lessons were 1 hour 20 minutes.* —Teacher

#### Content Coverage

According to teachers’ lesson evaluations, all 10 lessons were delivered as planned. However, teachers observed that students struggled to grasp the concepts in 2 lessons: random allocation (randomly created groups) and random error (large-enough groups) (high confidence).

According to teacher’s lesson evaluations, all 10 lessons were delivered as planned.

*The only issue I think you should have been, the only parts that were difficult to teach were the 2. The large enough and the randomly created groups.* —Teacher

In the teachers’ interviews, they reported that they largely taught the content as outlined in the respective lesson plans but with some adaptations to help students understand the content. The adaptations included replacing the preset examples with more familiar ones that teachers deemed more appropriate. Also, teachers reported using Swahili to explain sections of the content due to low English proficiency among most of their students (moderate confidence).

*Most of my students had “language marasmus” because a student with 121 marks out of 500, a student who cannot even speak English and you know the lessons were in English.* —Teacher

Data from lesson evaluation forms and from interviews indicate that almost all students enrolled at the onset of the trial attended all the lessons and completed the Critical Thinking about Health test used to measure outcomes. The few who missed lessons were either sick or had been sent home to collect school fees (high confidence).

#### Content Delivery Teaching Strategies and Methods

Teachers reported using all the prescribed teaching strategies, such as role-play, group discussion, buzz groups, response cards, question and answer, and lecture, from the IHC resources to deliver the respective lesson plans (projector-based and blackboard-based versions) (high confidence).

In interviews, all teachers and students from schools using the projector version stated that the slides made lessons more engaging and facilitated both understanding and note-taking. However, power outages posed a challenge, which they addressed by rescheduling lessons once power was restored. Teachers using the blackboard version reported that it was well-received by students. However, they felt they spent more time writing notes on the blackboard, as students with low English proficiency struggled to follow the lesson and take notes unless the teacher wrote the content on the board (moderate confidence).

### Factors That Facilitated Implementation

#### Teachers’ Confidence to Teach the Lessons

Teachers found the training workshop helpful in preparing them for the delivery of IHC lessons. As shown in Table 3, the training evaluation data indicate that 71. 8% and 28.2% of teachers rated the training overall as either excellent or good, respectively. The majority of teachers reported that the training enhanced their understanding of IHC key concepts and lessons and how to teach it effectively.

**TABLE 3.** Teachers’ Assessments of the Teacher Training in Informed Health Choices Intervention Lesson Observations, Kenya

	**Responses, No. (%)** **(N=39)**		
	**Neutral**	**Agree**	**Strongly Agree**
The training gave me general understanding of critical thinking about health.		3 (7.7)	36 (92.3)
The training gave me a clear overview and flow of all lessons.		9 (23.1)	30 (76.9)
I can navigate through the website, and I know where I can find all that I need.	2 (5.2)	8 (20.5)	29 (74.3)
Now I understand all teaching strategies relevant for teaching critical thinking about health.		12 (30.8)	27 (69.2)
The training gave me teaching tips that I need to consider while teaching the lessons.	1 (2.6)	8 (20.5)	30 (76.9)
I am confident that I understand and can teach all 10 lessons.	1 (2.5)	13 (33.4)	25 (64.1)
The training met my expectations.	1 (2.6)	12 (30.7)	26 (66.7)
I will be able to apply the knowledge learned.		5 (12.8)	34 (87.2)
		**Good**	**Excellent**
Overall rating of the training.		11 (28.2)	28 (71.8)

The evaluation data further showed that 97.5% of the teachers felt confident about teaching all the lessons. However, after the delivery of the lessons to the students, all the interviewed teachers said that they felt less confident about teaching the 2 lessons about random error and random allocation, which were confusing to them. They lacked words to explain the concepts to students fully, had inadequate comprehension of the concepts themselves, and felt there were too many statistics. They felt that the approach to tackling the lessons was not adequate. This finding is consistent with our observations during the training workshop, where some of these questions were raised about these 2 lessons. For example, teachers grappled with questions such as “How large is large enough?” (high confidence).

The teachers indicated there were too few days of training and not enough time for clarification and discussion, especially of the concepts and lessons about research (randomly created groups and large-enough groups). They advised that future training could be extended to 4 days, as described in this excerpt from an interview with 1 of the teachers (high confidence).

*The training was too short, especially when it comes to lesson 6 and 7, I tell you, I didn’t get anything.* —Teacher

#### Teachers’ Attitude

Interviews with teachers indicate that teachers found the content relevant, and they had a positive attitude toward the lessons. They suggested the content helped students to appraise information, avoid being misled by misinformation, and make informed decisions (high confidence).

*Lessons were very relevant for the learners…this program is very good. Personally, I feel it is very good because it will help us solve very many problems, because nowadays if you listen to even news, then people are taking some actions innocently not knowing what is the outcome.* —Teacher

#### Teaching Strategies

We observed that teachers used different teaching approaches that allowed students to learn from each other. Most interviewed students agreed with this observation and said they preferred the IHC lessons because the teacher’s attitude and approach encouraged discussion (high confidence).

Students pointed out teaching strategies such as question-and-answer sessions, group discussions, and response cards were well-suited for learners across different levels of academic performance. This allowed them to learn from each other (moderate confidence).

*In this lesson, people were arguing, each person defended his opinion to be right, people were raising their hands, people were making fun, everyone was enjoying.* —Student

#### Students’ Academic Performance, Reading Ability, and English Proficiency

We noted divergent views between teachers and students regarding the relationship between students’ academic performance and their understanding of IHC key concepts. The student interviews indicated that keen students could understand the concepts irrespective of their academic performance. However, teachers said understanding the concepts was easier for students who were high-to-average academic performers with good reading skills and English proficiency (high confidence).

#### Students’ Perceived Value and Motivation to Learn

Almost all students said that, unlike other subjects, IHC content dealt with common situations in their daily lives. Students said they were motivated to learn so that they could help teach their peers and family how to appraise health claims and make informed choices (high confidence).

*What I learned in these lessons was helping me to know more, so I could go and teach others and I could stop them from doing things that they do without thinking first.* —Student

Some students mentioned that the lessons helped them acquire the confidence to defend their contributions in arguments or debates (moderate confidence).

*What motivated me was the confidence of standing in front everyone while answering questions and feeling proud that everyone was listening to me.* —Student

Others thought the lessons helped their career aspirations, such as becoming doctors or researchers.

*Because my dream is to become a doctor, it can help me to know about health actions before maybe I treat someone or attend to someone.* —Student

Most students said at the start of the lessons they had viewed the lessons as not motivating and a waste of time because learning objectives were not included in the curriculum, not included in the national examination, and that some of the lessons were taught during their free time. Almost all students interviewed said that they did not want to sacrifice their free time in the beginning but later agreed to attend additional classes to complete the lessons (high confidence).

### School- and Policy-Level Factors

All interviewed teachers said the administration supported implementation of the IHC intervention. This included support for scheduling time within and outside the timetable for teaching the lessons and printing services (high confidence).

The curriculum developers, subcounty education officers, and representatives of the Teacher’s Service Commission said that they authorized the lessons because of their perceived relevance and long-term benefits to the students (high confidence).

*The research will go a long way in helping the future generations in critical thinking … When you look at this particular program, it is touching much on the life of student as they are going to grow. Then there are some aspects of life they should be developing at an early age*. —Curriculum Developer

### Beneficial Effects

#### Application of the Key Concepts in the Context of Health

After the intervention, most interviewed students demonstrated that they could now identify and recognize health claims, ask questions about claims, make informed choices, and defend their health choices. In the interviews, students indicated they felt more confident discussing beliefs about health actions than before the lessons (high confidence).

After the intervention, most interviewed students demonstrated that they could now identify and recognize health claims, ask questions about claims, make informed choices, and defend their health choices.

*I told my mum that she… she should stop forcing me to take medicine from the pharmacy (okay). I should go direct to the hospital. Now [we’re] questioning a lot of instructions given by our parents. If you are feeling unwell you are told to take some pain killer to relieve the pain, but you’re asking why. How do you know that the pain killer will relieve my pain? How do you know what I am suffering from? So why did you take those [painkillers]?* —Student

Some students said that if they were not convinced by the basis for a claim, they would refuse to take the proposed health action or treatment (moderate confidence).

Some parents said they were impressed with their children’s improved aptitude and confidence to critique and challenge some of their decisions and that they would accept their children’s choices, such as going to the hospital to get a correct diagnosis and prescription (high confidence).

*The lesson you have brought to the children has opened our eyes and now we know that we should not just take any drug without it being tested.* —Parent

We noted that nearly all interviewed students shared at least 1 example of how they applied the key concepts. The concept was almost always 1 of these 3:

- Weigh the advantages and disadvantages of health actions.
- Personal experiences or anecdotes alone are an unreliable basis for most claims.
- New and expensive treatments may not be better than available alternatives.

#### Application of Key Concepts Outside of Health

We noted from interviews and classroom observations that students applied some key concepts outside of health (high confidence).

*Then he advised me to go with him [to a] party… Then … I tried questioning him so that I can know where we were going. But when I looked at whatever he told me, I looked at the advantages and disadvantages. I tried to convince him. I totally disagreed with him… So, I think after that it’s unfortunate that all of his friends who went to the party had a road accident and… so it’s good to make decisions.* —Student

#### Benefits to Teachers

We found that teachers also applied the key concepts in their daily lives. For example, they reported using the concepts to recognize unreliable health claims. They shared examples of how they were now able to recognize how claims on social media, TV adverts, and from their colleagues or friends could mislead them. However, some teachers said reconsidering long-held beliefs was sometimes challenging, especially beliefs about herbal treatments based on personal experience (high confidence).

## SUGGESTIONS FOR SCALING-UP THE INTERVENTION IN KENYA

### Incorporate Lesson Objectives Into the Curriculum

Students, teachers, curriculum developers, and policymakers in education suggested incorporating the IHC lesson objectives into the Kenya primary and secondary school education program. The teachers added that the incorporation should use a spiral approach, starting with simple key concepts in lower classes and introducing more complex ones (e.g., research-based concepts) in the upper levels (moderate confidence).

The curriculum developers and some teachers noted that the IHC lesson objectives are related to the goal of the new Kenyan competency-based curriculum that seeks to develop students’ critical thinking skills and other higher-order thinking skills. The curriculum developers suggested subjects in the new curriculum that could provide opportunities to implement the lessons within the curriculum. These included life skills, health education, biology, chemistry, and home science. Policymakers and curriculum developers suggested the content should also be taught in post-secondary education, especially in medical schools (high confidence).

### Include Informed Health Choices Training in Teacher Training

Nearly all the teachers that we interviewed said their prior in-service training addressed concepts such as critical thinking, reasoning, and questioning skills but that it did not explicitly cover concepts such as the key concepts in the IHC resources. The teachers, school principals, and curriculum developers suggested including the IHC training in the in-service training. They also suggested that training more than 1 teacher per school would ease the burden of relying on 1 teacher and would enable teaching of the lessons to more than 1 class (high confidence).

### Develop Student Textbooks

Teachers and students noted that besides the digital IHC resources, printed textbooks are needed for students. They said this will be used by students as reference books and can be accessed anytime because not all students have phones or laptops to access them digitally (high confidence).

### Expand Coverage Outside School

Some teachers and students suggested that IHC digital resources, such as short videos and audio versions, should be developed and posted on platforms such as YouTube, which could be accessed by young people and the public in and outside school (moderate confidence).

### Involve Stakeholders

Teachers, parents, and county education authorities emphasized the importance of engaging parents and other key stakeholders in implementing interventions like the IHC secondary school intervention. The curriculum developers noted that parents could contribute to learning and application of learned skills at home. Some of the parents interviewed also indicated that they would like to know more about school programs and how to participate (high confidence).

## DISCUSSION

We found that the IHC secondary school intervention was implemented as planned, with minimal adaptations to fit students’ and teachers’ needs. Factors that facilitated the effective implementation included the teacher’s training workshop, the perceived value of the intervention by multiple stakeholders, and support from education officials and school management. Time constraints and teachers’ heavy workloads were the major factors that adversely affected implementation of the intervention. Both teachers and students were able to apply the key concepts in their daily lives.

Teaching the lessons took more time (on average, 50–120 minutes) than the 40 minutes for a standard lesson that we suggested.^18^ It is possible that teachers needed more time to teach the lessons because they were teaching the material for the first time. Another reason may be that students were encouraged to participate actively in the lessons rather than sitting through them passively. If the IHC lessons are incorporated into the curriculum, it may be necessary to increase the time allocated for teaching the lessons. The parallel process evaluations in Rwanda and UGANDA found variation in the time teachers used to teach the lessons.^26^^,^^27^ The process evaluation in Rwanda found that teachers used approximately 40 minutes (42–46 minutes), while the Ugandan process evaluation found that teachers used an average of 69–80 minutes for the lessons.^26^^,^^27^ The primary school lessons in Uganda took 80 minutes on average.^18^

The teachers were able to teach 8 of the lessons without difficulties. However, both teachers and students reported difficulties with the 2 lessons that focused on assessing the reliability of research and included the following key concepts.

- In a comparison between health actions, important differences (other than the health actions) between comparison groups can be misleading. Randomly creating groups makes sure groups of people are as similar as possible at the start of a comparison and avoids unknown differences.
- If a comparison between health actions is too small, we cannot be sure that the results reflect a true difference (or lack of difference) between the effects of the different health actions. The results could just be by chance.

Research and research-based concepts such as these are not normally taught in primary or lower secondary school levels in Kenya and may be taught in later years of secondary school or in higher education. The adjusted differences in the proportion of students who understood and could apply these concepts were 22.8% (95% confidence interval [CI]=16.6, 29.0) and 15.8% (95% CI=11.4, 20.1), respectively.^21^ These differences were as large or larger than the differences for other key concepts included in the lessons. Taken together, these findings suggest that it may be necessary to improve how all the lessons are taught to help ensure that all students understand and are able to apply the concepts. These improvements might include allocating more time to the lessons as previously noted and particularly to those 2 lessons, allocating more time to the teacher training, revising how the lessons are taught, introducing these concepts in upper secondary school, and introducing a spiral curriculum over all 4 years of secondary school. A spiral curriculum, as suggested by teachers that we interviewed, would introduce concepts to students early and cover these concepts repeatedly, with increasing degrees of difficulty and reinforcement of previous learning.^28^^,^^29^

Factors that potentially facilitated the effective implementation of the intervention included the teacher training workshop, the perceived value of the lessons by students, teachers, and other stakeholders, and support from the school administration. These factors are consistent with findings of the process evaluation of the IHC primary-school intervention.^18^ The perceived value of the lessons reflects people’s recognition that they need critical thinking skills to be able to cope with the overabundance of health claims, a need that intensified with the very large amounts of accurate and inaccurate information circulating during the COVID-19 pandemic.^30^ The perceived value of the lessons may also be attributable to the human-centered approach we used to design the intervention.^20^ From August 2019 to April 2022, we engaged in an iterative process of generating ideas, creating prototypes, and testing them, all while prioritizing the needs and experiences of users and stakeholders. Additionally, we conducted a systematic review of studies on teaching critical thinking to identify effective strategies that could enhance our resources, aiming to learn from past efforts in this area.

The perceived value of the lessons reflects people’s recognition that they need critical thinking skills to be able to cope with the overabundance of health claims.

The barriers to effective implementation of the intervention that we identified are also consistent with those identified in the process evaluation of the IHC primary-school intervention.^20^ If the IHC lessons are not included in the national curriculum, school timetables, and national exams, it will be hard to scale up the intervention. The competencies and dispositions targeted in this intervention align closely with those in the existing competency-based curriculum, and this has sparked interest in the materials. We have also identified potential school subjects into which these concepts could be integrated.

Both the trial and the process evaluation findings found that at least some students were able to apply some of the concepts. However, teachers said that students found it difficult to follow the lessons, particularly those students with low English proficiency. Translation of the resources from English to local languages may have helped students understand the lessons and increased the proportion of students able to apply the key concepts.

### Strengths and Limitations

A key strength of this process evaluation is the use of mixed methods, which allowed for triangulation of data gathered through different methods. This facilitated validation and assessing the reliability of the findings through cross-verification. Another strength is that we systematically assessed confidence in our findings using a modified GRADE-CERQual approach.

A key limitation of this study comes from using an inductive approach, guided by the previous framework developed by our team to explore and understand factors that facilitated or hindered the implementation of the IHC primary school intervention.^18^ This approach had the potential to skew the data collection and analysis. We mitigated this risk by being open to including other emerging themes and factors and adapting the framework. Another key limitation is that our research team both developed and evaluated the intervention, which might have biased us toward positive findings. We mitigated this by having independent researchers who were not involved in developing or evaluating the intervention help collect and analyze the data and grade confidence in the findings of this process evaluation.

## CONCLUSION

This study suggests that the IHC secondary school intervention was implemented largely as intended, with minimal adaptation to fit the needs of teachers, students, or schools. Teachers, students, and other stakeholders value learning how to think critically about health, and this facilitated the implementation of the intervention. Both students and teachers demonstrated the application of the key concepts included in the lessons to health claims and choices and to other types of claims and choices. Scaling up the intervention is likely to depend on the lessons being included in the national curriculum.

## Supplementary Material

GHSP-D-23-00485-Supplement.pdf

## Notes

**Peer Reviewed**
