# Supplementary material for: Process Evaluation of Teaching Critical Thinking About Health Using the Informed Health Choices Intervention in Kenya: A Mixed Methods Study
Source: Glob Health Sci Pract. 2024 Dec 20;12(6):e2300485. doi: 10.9745/GHSP-D-23-00485 (PMC11666096; doi:10.9745/GHSP-D-23-00485)
Supplement: GHSP-D-23-00485-Supplement.pdf [file GHSP-D-23-00485-Supplement.pdf]

Supplement to: Chesire F, Oxman AO, Kaseje M, et al. Process evaluation of teaching critical thinking about health using the Informed Health Choices Intervention in Kenya: a mixed methods study. *Glob Health Sci Pract*. 2024;12(6):e2300485. https://doi.org/10.9745/GHSP-D-23-00485

SUPPLEMENT 1. GUIDELINE FOR REPORTING EVIDENCE-BASED PRACTICE EDUCATIONAL INTERVENTIONS AND TEACHING (GREET) CHECKLIST[1]

BRIEF NAME

1. **Intervention:** Informed Health Choices (IHC) secondary school intervention

The intervention was compared to routine practice (teaching according to the national lower-secondary school curriculum without intervening).

WHY this educational process

2. **Theory:** The IHC secondary school resources are based on the *IHC Key Concepts* framework. The framework includes concepts (principles) that people should understand and apply when deciding whether to believe a claim about the effects of health actions (things that people do to care for their health or the health of others) and what to do. [2,3] The framework is based on evidence of the importance of the included concepts, [4,5] logic, feedback, other relevant frameworks, [6] and adaptation of the IHC Key Concepts to other types of interventions such as educational, environmental, and policing interventions. [7]

The resources were developed by the investigators between 2020 and 2022 using human-centred design methods. [8] This included cycles of idea generation and prototyping, piloting with observation, user-testing with teachers and students, and feedback from teachers, students, and curriculum developers in Kenya, Rwanda, and Uganda, and an international advisory group. The aim of the design process was to ensure that teachers and students find the resources to be engaging, useful, and easy to use.

The teaching strategies used in the resources were based in part on an overview of systematic reviews of teaching strategies, [9] and draw on several educational theories. These include social constructivist theory (which postulates that learning can be maximized through well-designed, intentional social interaction with other learners), [10] the theory of active student response (which postulates that learning is enhanced by high levels of active student response), [11] and the elaborative retrieval hypothesis (which postulates that the search for correct answers on practice tests or quizzes results in multiple retrieval routes which aid later recall). [12]

3. **Learning objectives:** The primary learning goal is for students to have a basic ability to think critically about health actions and understand why this is important. They should be able to recognise claims about the effects of health actions and assess some of those claims. They should understand why it is important for them that researchers study the effects of health actions and recognise two key features of reliable comparisons of health actions. They should recognise that health actions can have both advantages and disadvantages and the importance of weighing the benefits and savings against the harms and costs when deciding what to do.

4. **Evidence-based practice content:** The resources focus on nine IHC Key Concepts that were prioritised by curriculum developers, teachers, and researchers in Kenya, Rwanda, and Uganda.[13]

WHAT

---

5. **Materials:** The IHC secondary school resources (*Be smart about your health*) are open access digital resources for lower-secondary school teachers. The 10 lessons are provided as lesson plans in two formats: for teachers who are using either a blackboard and or a projector in the classroom. The aim is for students to learn to think critically about health claims and choices. The resources were made available to schools in the intervention group. Teachers in those schools downloaded the resources to a computer or smartphone and delivered the lessons. Schools in both the control and intervention group continued teaching the national curriculum, which did not include teaching critical thinking about health. No additional materials were provided to the control schools.

Each Lesson includes an introduction, an activity, and a wrap-up. The introduction includes the key messages from the previous lesson, a question about the previous lesson, and what this lesson is about. The activity is designed to help students achieve the learning goals. The wrap-up includes a question about what was learned, the key messages for the lesson, a homework assignment, if there is one, and what the next lesson is about. Lessons 5 and 10 include quizzes and discussions of application of what students learned in their daily lives.

For each of the 10 lessons there is an overview and background for teachers. The overview includes learning goals, key terms introduced in the lesson, and the main teaching strategies used in the lesson. The background includes a description of what the lesson is about and if relevant, common misunderstandings and closely related content that is not covered in the lesson.

In addition, there is a teachers' guide, materials for teacher training workshops, information about how to use the resources (help), optional printouts (PDFs) for teachers and students, and a glossary. Teachers were provided with binders with printouts at the training workshops.

6. **Educational strategies:** Key strategies used across lessons included guided note taking, small group discussion, use of response cards,[11] homework, use of a standard lesson structure, setting objectives and providing feedback, and multimedia design. Other strategies used in some of the lessons include concept cartoons, inquiry-based instruction, and role play.

7. **Incentives:** The incentive for teachers and students was the value they perceived in learning to think critically about health actions. Teachers at schools without Internet access were reimbursed for the cost of downloading the resources and any other costs related to participation in the trial. They were not paid for participating in the trial and there were no

other financial incentives for the schools, head teachers, teachers, or students. The evaluation administered at the end of the school term did not count towards the students' school marks or assessment of the teachers or schools.

WHO PROVIDED

8. **Instructors**: The head teacher at each participating school selected a teacher of a relevant subject (e.g., biology) for year-1 or year-2 of lower-secondary school. The teachers were invited to a 2-3-day workshop to introduce them to the resources and the learning content. The training was facilitated by other teachers who had participated in one of the teacher networks that helped to develop the resources or who piloted use of the resources. The facilitators were provided with presentations and other materials for the workshops, and they reviewed the material and plans for the workshops with the research teams prior to the workshops.

HOW

9. **Delivery:** The 10 lessons were delivered by the teachers during regular classroom time or, if necessary, outside of regular classroom time. They could use a computer, smartphone, or printouts when delivering the lessons. Depending on what equipment was available to the teachers, they delivered the lessons to students using only a blackboard or using a projector and slide presentations that are included in the digital resources. The number of students in a class varied.

WHERE

10. **Environment:** Representative samples of schools were recruited, including rural and urban schools. The conditions in the schools varied. Details of the contexts in each of the three countries can be found in reports of the context analyses undertaken prior to developing the resources. [14-16]

WHEN and HOW MUCH

11. **Schedule:** The 10 lessons were taught in a single school term. Each school decided how to fit the lessons into the schedule for that term.

12. **Amount of time**: Each lesson is designed to be delivered in a single period (40 minutes). The students were encouraged to collect and assess claims about the effects of health actions outside of class and to discuss claims with their families and friends. The teachers needed up to 30 minutes to prepare for each lesson.

PLANNED CHANGES

13. **Adaptation**: No specific adaptation was required, but teachers were able to adapt the lessons, for example by using different or additional examples or editing the presentations.

UNPLANNED CHANGES

14. **Modifications**: As part of the process evaluations, teachers were asked to complete an evaluation form after each lesson, including information about changes they made to the lesson plan, and each teacher was observed for one lesson. No feedback was given to the teachers during the trial.

HOW WELL

15. **Attendance:** The teachers were asked to record attendance for each lesson. Students were encouraged to attend all lessons by telling them when the next lesson would be and its learning goals. The lessons were designed to appeal to students and to make clear the relevance and importance of the learning goals.

16. **Fidelity**: We will explore the extent to which the lessons were delivered as planned in the process evaluation, based on the evaluation forms completed by teachers after each lesson, observations of their teaching a lesson, and interviews with teachers and students.

17. **Delivery schedule**: The teachers were asked to record when each lesson was taught, the duration of each lesson, and whether all the lesson were completed as planned.

References

1. Phillips AC, Lewis LK, McEvoy MP, et al. Development and validation of the guideline for reporting evidence-based practice educational interventions and teaching (GREET). *BMC Med Educ* 2016; **16**: 237.
2. Oxman AD, Chalmers I, Austvoll-Dahlgren A, Informed Health Choices Group. Key Concepts for assessing claims about treatment effects and making well-informed treatment choices. *F1000Res* 2019; **7**: 1784.
3. Oxman AD, Chalmers I, Dahlgren A, Informed Health Choices Group. Key Concepts for assessing claims about treatment effects and making well-informed treatment choices. Version 2019. *IHC Working Paper* 2019.
4. Oxman AD, Chalmers I, Dahlgren A, Informed Health Choices Group. Key Concepts for Informed Health Choices: a framework for enabling people to think critically about health claims (Version 2022). *IHC Working Paper* 2022.
5. Oxman AD, Chalmers I, Dahlgren A. Key Concepts for Informed Health Choices: Where's the evidence? *F1000Res* 2022; **11**: 890.
6. Oxman AD, Martinez Garcia L. Comparison of the Informed Health Choices Key Concepts to other frameworks that are relevant to learning how to think critically about treatment claims, comparisons, and choices: protocol for a mapping review. *IHC Working Paper* 2018.
7. Aronson JK, Barends E, Boruch R, et al. Key concepts for making informed choices. *Nature* 2019; **572**(7769): 303-6.

8. Rosenbaum SE, Moberg J, Chesire F, et al. Teaching critical thinking about health information and choices in secondary schools: human-centred design of digital resources. *F1000Res* 2023.
9. Oxman AD, Nsangi A, Martinez Garcia L, et al. The effects of teaching strategies on learning to think critically in primary and secondary schools: an overview of systematic reviews. *Review of Education* Submitted 20 January 2023.
10. Igel C. Cooperative learning. Denver, CO: McREL International, 2010.
11. Randolph JJ. Meta-analysis of the research on response cards: effects on test achievement, quiz achievement, participation, and off-task behavior. *J Posit Behav Interv* 2007; **9**(2): 113-28.
12. Pan SC, Rickard TC. Transfer of test-enhanced learning: meta-analytic review and synthesis. *Psychol Bull* 2018; **144**(7): 710-56.
13. Agaba JJ, Chesire F, Mugisha M, et al. Prioritisation of Informed Health Choices (IHC) Key Concepts to be included in lower-secondary school resources: a consensus study. *PLoS One* 2023: in press.
14. Mugisha M, Uwitonze AM, Chesire F, et al. Teaching critical thinking about health using digital technology in lower secondary schools in Rwanda: A qualitative context analysis. *PLoS One* 2021; **16**(3): e0248773.
15. Ssenyonga R, Sewankambo NK, Mugagga SK, et al. Learning to think critically about health using digital technology in Ugandan lower secondary schools: a contextual analysis. *PLoS One* 2022; **17**(2): e0260367.
16. Chesire F, Ochieng M, Mugisha M, et al. Contextualizing critical thinking about health using digital technology in secondary schools in Kenya: a qualitative analysis. *Pilot and Feasibility Studies* 2022: Forthcoming.

Supplement to: Chesire F, Oxman AO, Kaseje M, et al. Process evaluation of teaching critical thinking about health using the Informed Health Choices Intervention in Kenya: a mixed methods study. *Glob Health Sci Pract*. 2024;12(6):e2300485. https://doi.org/10.9745/GHSP-D-23-00485

SUPPLEMENT 2. FRAMEWORK FOR FACTORS THAT COULD AFFECT THE IMPLEMENTATION, IMPACT, AND SCALING UP OF THE SCHOOL RESOURCES

Domain	Factors and sources	Explanation
Teachers	Skills and competencies	Teacher's education and experience in relation to the lessons being taught.
	Understanding of the content being taught	Teacher's understanding of the content.
	Sufficient training	The extent to which the teachers received sufficient training in teaching the lessons
	Self-efficacy	Teacher's confidence in teaching the lessons.
	Fit to the teacher's teaching style and context (eg, class size)	Teachers' comfort with the instructions or ability to adapt the instructions to their style and context.
	Attitudes	Teachers' attitude towards new material (change), science, critical thinking and independent thinking by pupils (or their role as authorities in the classroom).
	Beliefs	Teachers' beliefs about the teaching methods or content (eg, what treatments work or the concepts).
	Emotions	Teachers' emotions, such as stress or anxiety.
	Motivation	Teachers' motivation to teach the material.
	Positive learning environment	Teachers' ability to create a positive learning environment; for example, encourage discussion, respond positively to questions, engage pupils.
Learners	Literacy	Learners' ablity to understand the material.
	Attendance	Learners' attendance or reasons for poor attendance (eg, long distance to school or inability to pay school fees).
	Motivation to learn	Learners' motivation to learn the new material.
	Attitudes	Learners' attitudes towards learning, towards authorities, towards science, towards critical thinking.
	Beliefs	Learners' beliefs about the content (eg, what treatments work or the concepts).

Supplement to: Chesire F, Oxman AO, Kaseje M, et al. Process evaluation of teaching critical thinking about health using the Informed Health Choices Intervention in Kenya: a mixed methods study. *Glob Health Sci Pract*. 2024;12(6):e2300485. https://doi.org/10.9745/GHSP-D-23-00485

Domain	Factors and sources	Explanation
	Home environment	The extent to which the learners's home environment encourages or discourages learning from the lessons.
	Differentiated instruction	The extent to which learners different learning needs are met.
	Peer influence	Positive or negative attitudes of other learners towards the material.
Teaching materials	Value of the material	The extent to which the materials are valued by the teachers and learners.
	Compatability with the curriculum	The extent to which the material fits with the rest of the curriculum and how it is taught.
	Appropriateness of the material	The extent to which the materials are relevant, challenging and engaging.
	Credibility of the material	The textent to which the teachers and learners perceive the material as credible.
School system and environment	Time constraints	The extent to which there is sufficient time to accommodate introducing the new material.
	Competing priorities	The extent to which other priorities for the school, teachers or pupils limit introducing the material (eg, preparing for exams).
	School organisation and management	The extent to which the school provides an environment that supports adoption of new subjects, material and teaching methods.
	School resources, particularly human resources	The extent to which the school has adequate resources to introduce the new materials (eg, human resources, student/teacher ratio, teacher workload, classroom space and classroom resources, such as blackboards and acoustics).
	Attitudes and beliefs of head teacher and other teachers	Attitudes or beliefs of colleagues that influence the teacher's interest in and ability to teach the material.
	Parent and community involvement	Parents' attitudes towards the new material or how things are done at the school.
	Regulation	Regulations (eg, Ministry of Education policies and regulations) that affect introducing the new material.

Supplement to: Chesire F, Oxman AO, Kaseje M, et al. Process evaluation of teaching critical thinking about health using the Informed Health Choices Intervention in Kenya: a mixed methods study. *Glob Health Sci Pract*. 2024;12(6):e2300485. https://doi.org/10.9745/GHSP-D-23-00485

Domain	Factors and sources	Explanation
	Political environment	Elements of the political environment that affect introducing the new material; for example, authoritarianism or teacher strikes.
	Bureaucracy	Bureaucratic arrangements that delay or limit introduction of the new materials, or facilitate introducing them.
	Incentives and disincentives	Incentives or disincentives to introduce the new materials for teachers or head teachers.

Framework for categories of beneficial outcomes

Domain	Factors and sources	Explanation
	Correct and necessary application of learning	correct or unnecessary application of a skill or knowledge learned from the intervention
	Healthy scepticism	Believe that the application of skills or knowledge learned from the intervention is possible or useless
	Cognitive consistency	Experience of inconsistent beliefs
	Benefit-based equity	Equity due to the distribution or size of a beneficial effect of the intervention
	Constructive discussion	Constructive argument between two or more parties
	Worthwhile use of time and resources	Well use of time or resources on the intervention that would be better spent on other activity

Supplement to: Chesire F, Oxman AO, Kaseje M, et al. Process evaluation of teaching critical thinking about health using the Informed Health Choices Intervention in Kenya: a mixed methods study. *Glob Health Sci Pract*. 2024;12(6):e2300485. https://doi.org/10.9745/GHSP-D-23-00485

SUPPLEMENT 3. CERQUAL ASSESSMENT

Summarised review finding	Methodological limitations	Coherence	Adequacy	Relevance	GRADE-CERQual assessment of confidence	Explanation of assessment
**Objective 1: Extent to which the *Be Smart about your Health* secondary school intervention was implemented as planned**						
Teachers had insufficient time (15-30 minutes) to prepare adequately for the IHC lessons were new and challenging to conceptualize and some lessons need more time.	Data from individuals and group interviews. No concerns regarding participant selection and data collection and analysis	Very minor concerns as data fits the review finding.	Adequate data from teachers and principals Very minor concerns 38 of 40 teachers completed some lessons within the intended time forty minutes	No concerns as majority of the data supports the findings	High confidence	The finding was graded high as there were very minor concerns on coherence and no concern on adequacy, relevance and methodological limitations
Teachers across schools were unable to complete teaching IHC lessons within the intended single standard 40 minutes' instead used more minutes varying from 50 to 120 minutes to achieve a lesson objectives.	Data from individuals and observations. No concerns regarding participant selection and data collection	No concerns as data fits the review finding	Data from 40 participants. Reasonable data. No concerns	No concerns	High confidence	Due to no concerns regarding coherence, relevance, adequacy and methodological limitations
Teachers completed teaching all the IHC lessons and achieved lesson objectives for eight of the ten IHC lessons. However, they said only a few students achieved the objectives for the lessons about random error and allocation	Data from individuals and groups. No concerns regarding participant selection and data collection	No concerns as data fits the review finding	Data from 40 participants. Reasonable data. No concerns	No concerns views from various teachers both in evaluation, key informants and FGDs	High confidence	The finding was graded high due to no concerns on methodological limitations, coherence, adequacy and relevance
Teachers reported using the IHC resource as it were although with minimal adaptations for example;i) used of local language (Swahili) instead of English,ii) used some relatable local examples instead of those provided in the lessons,	Data from individuals and group discussions. No concerns regarding participant selection and data collection	No concerns as data fits the review finding	Data from 18 participants, reasonable thick data with explanations	Moderate relevance as teachers used examples not outlined in the material	Moderate confidence	The finding was graded moderate due to no concerns on methodological limitations, but with minor concerns on coherence and adequacy and moderate concerns on relevance
Almost all students that were enrolled in the project attended all ten IHC lessons and completed the CHT test. The few that missed some lessons were either sick or were at home to collect school fees	Data from individuals and group interviews and lesson evaluation No concerns regarding participant selection and data collection	No concerns as data fits the review finding	Data from 27 participants. Reasonable data across data sources.	No concerns as data come from different teachers, students and teachers evaluation	High confidence	The finding was graded high due to no concerns regarding methodological limitations, relevance and coherence and minor concerns on adequacy

Supplement to: Chesire F, Oxman AO, Kaseje M, et al. Process evaluation of teaching critical thinking about health using the Informed Health Choices Intervention in Kenya: a mixed methods study. *Glob Health Sci Pract*. 2024;12(6):e2300485. https://doi.org/10.9745/GHSP-D-23-00485

Summarised review finding	Methodological limitations	Coherence	Adequacy	Relevance	GRADE-CERQual assessment of confidence	Explanation of assessment
All teachers said the teaching strategies were familiar, easy to adapt, and the strategies were congruent with the strategies prescribed for teaching the new Kenya competency education curriculum. They added that response cards and group discussions provided opportunities for them to learn from each other.	Data from individuals and group interviews,. No concerns regarding participant selection and data collection	No concerns as data fits the review finding	Data from …Explanations were provided. Reasonable thick data. Minor concerns	No concerns	High confidence	This was due to no concerns on relevance, adequacy, coherence and methodological limitations
All students from the schools assigned to use the projector version enjoyed visual delivery of the lessons, sighting that visual lessons facilitated easy understanding of the content and also eased copying notes.	Data from student interviews. No concerns regarding data collection	No concerns as data fits the review finding	Data….participants. Explanations were provided	Minor relevance as teachers reported some visuals/images were not clear	Moderate confidence	This is due to no concerns on methodological limitations, minor concerns on coherence, adequacy and relevance
**Objective 2: Factors that affected implementation, impact, and scaling up of the school resources**						
All teachers thought the workshop was helpful but advised that future training periods can be extended to four days to allow time for discussions of complex concepts.	Data from individuals and group interviews. No concerns regarding selection of participants and data collection used	No concerns as data fits the review finding	Explanations were provided from all teachers. Reasonable thick data from the data sources	No concerns as majority of the data supports the findings	High confidence	The finding was high with no concerns on its coherence, relevance, and methodological limitations
Teachers said that they felt less confident about teaching two lessons (random error and allocation).	Data from individuals and group interviews No concerns regarding selection of participants and data collection used	No concerns as data fits the review finding	Explanations were provided from most teachers from interviews and lesson evaluations	No concerns as majority of the data supports the findings	High confidence	The finding was high with no concerns on its coherence, relevance, and methodological limitations
All teachers after the training stated high confidence to teach the lesson but felt less confident to teach two concepts – 'large enough' and 'randomly selected.	Data from individual and group interviews. No concerns regarding participant selection and data collection	Very minor concerns as data fits the review finding.	Data from 14 participants. Rich data with reasonable explanations. Very minor concerns	Minor concerns	High confidence	This is due to minor concerns on relevance
Teachers indicate that teachers found the content relevant had a positive attitude towards the lessons.	Data from individuals interviews and group. No concerns regarding data collection and participant selection	No concerns as data fits the review finding	Data from... participants. Rich data with reasonable explanations. No concerns	Data across all interviews. Very minor concerns	High confidence	This is due to very minor concerns on relevance and no concern on adequacy, methodological limitations and coherence

Supplement to: Chesire F, Oxman AO, Kaseje M, et al. Process evaluation of teaching critical thinking about health using the Informed Health Choices Intervention in Kenya: a mixed methods study. *Glob Health Sci Pract*. 2024;12(6):e2300485. https://doi.org/10.9745/GHSP-D-23-00485

Summarised review finding	Methodological limitations	Coherence	Adequacy	Relevance	GRADE-CERQual assessment of confidence	Explanation of assessment
Teachers created opportunities for students to ask questions, and most students acknowledged the teacher's positive attitude and approach that encouraged class discussions, especially responding to the questions in a friendly manner.	Data from individuals interviews and group. No concerns regarding data collection and participant selection	No concerns as data fits the review finding	Data from... participants. Rich data with reasonable explanations. No concerns	Data across all interviews. Very minor concerns	High confidence	This is due to very minor concerns on relevance and no concern on adequacy, methodological limitations and coherence
The student's interviews noted that keen students would understand those concepts irrespective of their academic performance. While teachers said understanding the concepts was easier for students who were generally high-to-average academic performers with good reading skills and English proficiency	Data from individuals interviews and group. No concerns regarding data collection and participant selection	No concerns as data fits the review finding	Data from... participants. Rich data with reasonable explanations. No concerns	Data across all interviews. Very minor concerns	High confidence	This is due to very minor concerns on relevance and no concern on adequacy, methodological limitations and coherence
Most students got motivated in learning the IHC because they found that IHC lessons were relatable to them and valuable to deal with real-life experiences.	Data from group interviews. No concern regarding data collection	No concerns	Data from 21 participants, minor concern.	Minor concerns.	High confidence	No concerns
Students were motivated to learn IHC lessons since they acquired confidence to stand before people and defend their contributions in arguments/debates.	Data from group interviews. No concern regarding data collection	Minor concerns	Data from 3 FGDs	Minor concern	Moderate confidence	Due to minor concerns on relevance and adequacy
However, almost all students were not willing to let go of their free time timetable (remedial, games time, weekend, night preps) at the beginning but later agreed thus they created additional classes outside the to complete the lessons.	Data from individual and group interviews. No concerns regarding data collection	No concerns as data fits the review finding.	Data from 34 participants. Reasonable thick data with explanations	No concern	High confidence	Due to no concerns regarding coherence, relevance, adequacy and methodological limitations
Teachers used different teaching approaches that allowed students to learn from each other. Most interviewed students agreed with this observation and said they preferred the IHC lessons because the teacher's attitude and approach encouraged discussion	Data from individuals and group interviews. No concerns regarding participant selection and data collection	No concerns as data fits the review finding	Data from …Explanations were provided. Reasonable thick data. Minor concerns	No concerns	High confidence	This was due to no concerns on relevance, adequacy, coherence and methodological limitations

Supplement to: Chesire F, Oxman AO, Kaseje M, et al. Process evaluation of teaching critical thinking about health using the Informed Health Choices Intervention in Kenya: a mixed methods study. *Glob Health Sci Pract*. 2024;12(6):e2300485. https://doi.org/10.9745/GHSP-D-23-00485

Summarised review finding	Methodological limitations	Coherence	Adequacy	Relevance	GRADE-CERQual assessment of confidence	Explanation of assessment
Almost all students across schools felt that the teachers met their different learning needs during the delivery of IHC lessons. According to the students they indicated that teachers used teaching strategies such as question and answer sessions, group discussions and buzz groups which suited and favored all learners of different academic performance levels (low, average, high) to learn	Data from group interviews. No concerns on data collection	No coherence		Moderate relevance	Moderate confidence	
All teachers indicated they received adequate support to implement the IHC program from their respective school administrations .	Data from individuals and group. No concerns regarding data collection	No concern	Data from 40 participants. Explanations were provided	very minor concerns	High confidence	This is due to very minor concerns on relevance and no concerns on adequacy and coherence
Data shows that across board teachers and students and stakeholders (principals and curriculum developers) regarded the IHC intervention as relevant. Additionally both teachers, principals and curriculum developers indicated that IHC lessons provides much needed skills and knowledge for students as they navigate adolescence stage where there are too much rumors from all over to appraise.	Data from individual interviews and group	No concern as data fits the findings	Data from 32 participants	Minor concerns indirectly reporting for students and teachers	Moderate confidence	Due to minor concerns on relevance and adequacy
**Objective 3: Perceived impacts of the IHC secondary schools' intervention on the intended outcomes for students**						
Most students interviewed said that they felt more confident to negotiate for treatments/health actions or other social-related actions/options better after learning IHC key concepts.	Data from group interviews, No concern on data collection	No concerns	Data from all participants in students and few parents. Rich data with explanations. Very minor concern	No concerns	High confidence	No concerns
Some students indicated a few instances where they were adamant or completely refused to take up proposed health actions/treatments or a social decision from either parents or friends based informed by the key concepts. A few students noted that parents appreciated for engaging with students in negotiating for health decisions and seeking answers to questions however, most parents said were not comfortable with	Data from group interviews	No concerns as data fits the findings	Data from students interviews with 6 parents	Minor concern as there is no much data for parents	Moderate confidence	Due to minor concerns on relevance

Summarised review finding	Methodological limitations	Coherence	Adequacy	Relevance	GRADE-CERQual assessment of confidence	Explanation of assessment
students asking and questioning health decisions.						
Nearly all students interviewed demonstrated correct application of the intended competencies and dispositions linked to IHC lesson goals.	Data from group interviews. No concerns on data collection	No concerns	Data from students FGDs across schools	No concerns	High confidence	No concerns
Nearly all students across schools stated that they believed the IHC key principles and appreciated that they were learning important knowledge and skills that would help them. They demonstrated that they were able to apply some of the key concepts to their daily lives and outside health	Data from group interviews. No concerns on data collection	No concerns	Data from students FDGs	No concerns	High confidence	No concerns
Students, teachers, curriculum developers and other policy makers in education indicated the need of working with key stakeholders most importantly the Ministry of Education, Teachers Services Commission Institute of Curriculum Development to ensure the IHC lessons are incorporated into the lower and upper levels curriculum.	Data from individuals and group interviews. No concerns	No concerns	Data from…participants. Rich data Very minor concerns	Minor concern.	Moderate confidence	Due to minor concerns on relevance
Most the teachers and curriculum developers indicated that the IHC content is compatible with the goals and aspirations of the new Kenya competency-based curriculum. They also thought that the new curriculum may provide appropriate opportunity for teaching the principles.	Data from individual interviews and group. No concern on data collection	No concerns	Data from 34 participants. Reasonable thick data with explanations	No concerns	High confidence	No concern
Teachers that we interviewed said their prior in-service training addressed concepts such as critical thinking, reasoning, and questioning skills, but that it did not explicitly cover concepts such as the key concepts in the IHC resources. The teachers, school principals, and curriculum developers suggested including the IHC training in the in-service training. They also suggested that training more than one teacher per school would ease the burden of relying on one teacher and would enable teaching of the lessons to more than one class	Data from group interviews, No concern on data collection	No concerns	Data from all participants in students and few parents. Rich data with explanations. Very minor concern	No concerns	High confidence	No concerns

Supplement to: Chesire F, Oxman AO, Kaseje M, et al. Process evaluation of teaching critical thinking about health using the Informed Health Choices Intervention in Kenya: a mixed methods study. *Glob Health Sci Pract*. 2024;12(6):e2300485. https://doi.org/10.9745/GHSP-D-23-00485

Summarised review finding	Methodological limitations	Coherence	Adequacy	Relevance	GRADE-CERQual assessment of confidence	Explanation of assessment
Teachers and students noted that besides the digital IHC resources, printed textbooks are needed for students. They said this will be used by students as reference books and can be accessed anytime since not all students have phones or laptops to access them digitally.	Data from individuals and group interviews	Very minor concerns	Data from …participants. Minor concerns	Minor concerns. No student data on this	High confidence	This is due to minor concerns on relevance and adequacy
Some teachers and students suggested that IHC digital resources such as short videos and audio versions should be developed, which could be accessed by young people and the public in and outside school. They suggested these could be posted on platforms such as YouTube.	Data from individuals and group interviews. No concerns	No concerns	Data from…participants. Rich data Very minor concerns	Minor concern.	Moderate confidence	Due to minor concerns on relevance
Teachers, parents, and county education authorities emphasized the importance of engaging parents and other key stakeholders in implementing interventions like the IHC secondary school intervention.	Data from individuals and group interviews	Very minor concerns	Data from …participants. Minor concerns	Minor concerns. No student data on this	High confidence	This is due to minor concerns on relevance and adequacy

Supplement to: Chesire F, Oxman AO, Kaseje M, et al. Process evaluation of teaching critical thinking about health using the Informed Health Choices Intervention in Kenya: a mixed methods study. *Glob Health Sci Pract*. 2024;12(6):e2300485. https://doi.org/10.9745/GHSP-D-23-00485

SUPPLEMENT 4. TEAM-REFLEXIVE METHODS AND STATEMENT

Background

Reflexivity involves researchers reflecting on and communicating their *a priori* values, views, experiences and beliefs about the topic of interest, as well as their context, and how they might influence the research (1, 2). Researchers often apply reflexivity individually, but there are benefits to also considering how the dynamics, structure and expectations of the research team may influence the research (3). In a team-reflexive process, members of the research team can discuss how their individual and collective perspectives, beliefs, and experiences might influence or have influenced the design and or conduct of the research, or their interpretation of the findings. This discussion can inspire reflections and facilitate constructive questioning of each other's assumptions. Our objective in undertaking a group reflexivity exercise was to identify important issues to consider and address in the analysis and reporting of the study findings. These are reported in a reflexivity statement herein.

Methods

Written reflections from the team

MO, SL, and AO developed and circulated four open-ended, reflexive questions to members of the research team (FC, RS, SL, AN, DS, SR, JM, AO, AF, NS, MM, MO, MK, AF) and asked them to write their responses. MO, SL, and AO also submitted responses. The questions were:

- What findings do I expect to come out of the process evaluations?
- How do I anticipate that the findings will contribute to the CHOICE project overall?
- How might I shape the process evaluations or my views of them, based on my beliefs (e.g., about the impacts of the intervention); background and previous research experiences (e.g., my disciplinary training); or hopes or concerns related to the CHOICE project?
- What are my concerns related to the CHOICE project, if any?

Research assistants were not included in this stage in order to keep the amount of feedback manageable. AN coded and analysed the written responses using thematic analysis methods (4).

Team discussions

All team members were invited to a structured team-reflexive discussion (AF, CH, MK, SL, JM, MM, AN, AO, MO, SR, DS, NS) in January 2023 during a period where researchers were engaged in data analysis for the process evaluations. The discussion lasted two hours and was facilitated by HMK. Findings from the analysis of the written reflections informed a discussion guide focused on the following themes:

- What findings do I expect to come out of the process evaluations?
- How might I shape the process evaluations or my views of them, based on my beliefs (e.g., about the impacts of the intervention); background and previous research experiences (e.g., my disciplinary training); or hopes or concerns related to the CHOICE project?
- How do I anticipate that the findings will contribute to the CHOICE project overall?

The research team members were invited to a second structured team-reflexive discussion in April 2023 (AF, CH, MK, SL, JM, MM, AN, AO, MO, SR, DS, NS). HMK and MO prepared the discussion guide based on on the topics covered in the first team discussion. HMK facilitated the discussion around the following themes:

- What are other concerns aside from implementation and sustainability (covered during first discussion)?
- Where do these concerns come from?
- What are the relationships between the project team members and how does that impact the research?

Parts of the first team discussion were recorded. The second team discussion was recorded, but the recording was destroyed before transcription. Two people observed and took notes for each discussion.

HMK drafted a team-reflexive statement with key issues that we identified and discussed. All authors agreed on the final statement.

HMK had responsibility for planning and leading the discussions, and drafting the statement, because she did not contribute to the development of the intervention, nor to planning or conducting the evaluation.

Analyzing our reflections

HMK used framework analysis to identify themes from the team discussions (5). HMK combined the analyses from the written reflections and group discussions using thematic analysis (4). The following is a reflexivity statement based on the results of the written responses and two structured team-reflexive discussions.

Reflexivity Statement

Background of researchers

Supplement to: Chesire F, Oxman AO, Kaseje M, et al. Process evaluation of teaching critical thinking about health using the Informed Health Choices Intervention in Kenya: a mixed methods study. *Glob Health Sci Pract*. 2024;12(6):e2300485. https://doi.org/10.9745/GHSP-D-23-00485

The research team consisted of 16 researchers who represent a wide array of methodological experience, involvement in the Informed Health Choices project, and geographic and cultural backgrounds. Most of the researchers (SL, AN, DS, SR, JM, AO, AF, NS, MM, MO, MK, AF) were involved in the development of Informed Health Choices primary school intervention, as well as the secondary school intervention (6). Four of the researchers are leading various components of the CHOICE project as part of their doctoral work (FC, MM, RS, MO). None of the researchers are teachers, educational specialists, curriculum developers, or otherwise involved with the development or implementation of school curricula.

The team consisted mostly of researchers with health-related backgrounds. It may have been advantageous to have included researchers with more expertise in education and curriculum development when designing the studies and interpreting the findings. However, we did seek feedback from a wide variety of stakeholders at all stages of the project.

Research team members who were responsible for engaging with stakeholders, collecting feedback during intervention development, as well as implementing and evaluating the intervention had relevant geographical and cultural backgrounds. This in-depth understanding of the research contexts may have improved the design of the educational resources and the conduct of the studies.

Expectations regarding the process evaluation findings

Most of the research team expected positive findings from the process evaluations. However, all team members also expected important implementation and sustainability challenges to emerge from the process evaluation, and some team members expected findings of potential adverse effects of the intervention to emerge. These expectations may have influenced how the findings were interpreted.

Concerns regarding the intervention and evaluation

Effects, scale-up, and sustainability of the intervention

Within the team there were varying perspectives regarding whether or how the intervention could be implemented beyond the research project. Given that these views varied, it is unlikely that they influenced the analysis of the findings.

Scope of the evaluation

The research team may have viewed the findings only within the scope of the project and not sufficiently explored how this project fits in with, or could be enhanced by, other research in the field. On the other hand, the practice approach used in this project allowed for identifying issues that could be addressed and improved upon in further research.

Researchers' relationship to the project and to the participants

The review team was responsible for both developing and evaluating (collecting data for) the intervention. This may have hindered honest and critical feedback from the research participants (e.g., teachers) in that they did not want to offend or disappoint the researchers. It may also have influenced how the research team has analysed the data: since they were responsible for developing the resources, they may interpret results in a more positive manner.

Dynamics within the research team

Team members' responsibilities (e.g., leading process evaluations or a review of adverse effects) could have influenced their interpretation of the data from the process evaluation. For example, some members could have leaned towards overstating positive findings and overlooking negative findings due to the time, energy, and resources they had invested in developing the intervention.

Given that the study took place largely during the COVID-19 pandemic, there were limited opportunities in the earlier phases of the project for face-to-face meetings or team-building events, within and especially across countries. Some team members noted that this may have impacted on team dynamics. During the last phase of the project (data analysis and planning the 1-year follow up) many of the project team members from different countries met regularly face-to-face, but in Europe (SR, JM, AO, MO), not the East-African contexts in which we conducted the research.

References

1. Downe S, Finlayson KW, Lawrie TA, Lewin SA, Glenton C, Rosenbaum S, et al. Qualitative evidence synthesis (QES) for guidelines: paper 1–Using qualitative evidence synthesis to inform guideline scope and develop qualitative findings statements. Health research policy and systems. 2019;17(1):1-12.
2. Olmos-Vega FM, Stalmeijer RE, Varpio L, Kahlke R. A practical guide to reflexivity in qualitative research: AMEE Guide No. 149. Medical teacher. 2022:1-11.
3. Barry CA, Britten N, Barber N, Bradley C, Stevenson F. Using reflexivity to optimize teamwork in qualitative research. Qualitative health research. 1999;9(1):26-44.
4. Braun V, Clarke V. Thematic analysis: American Psychological Association; 2012.

Supplement to: Chesire F, Oxman AO, Kaseje M, et al. Process evaluation of teaching critical thinking about health using the Informed Health Choices Intervention in Kenya: a mixed methods study. *Glob Health Sci Pract*. 2024;12(6):e2300485. https://doi.org/10.9745/GHSP-D-23-00485

5. Ritchie J, Spencer L, O'Connor W. Carrying out qualitative analysis. Qualitative research practice: A guide for social science students and researchers. 2003;2003:219-62.
6. Nsangi A, Semakula D, Rosenbaum SE, Oxman AD, Oxman M, Morelli A, et al. Development of the informed health choices resources in four countries to teach primary school children to assess claims about treatment effects: a qualitative study employing a user-centred approach. Pilot and Feasibility Studies. 2020;6(1):1-15.
